# Elasticity as a Conservative Force in the Animal Organism

**Published:** 1887-04

**Authors:** T. Wesley Mills

**Affiliations:** Professor of Physiology, McGill University, Montreal


### Art. XI.—ELASTICITY AS A CONSERVATIVE FORCE IN THE ANIMAL ORGANISM.

BY T. WESLEY MILLS, M. A., M. D.,
*Professor of Physiology, McGill University, Montreal.*

Elasticity is a conservative property even in unorganized and inanimate bodies; but in living organisms it plays a part the importance of which amid the strains and complexities of existence we may guess at, but cannot adequately estimate.

The elasticity of the tissues gives the youthful form their firmer set, greater capacity to resist the effects of strains, falls, etc., adds to the beauty of form, and probably has much to do with high spirits. While all the tissues are more or less elastic, there is every degree of variation.

The elasticity of muscle is especially important and has been most carefully studied. The fact that this property is perfect up to a certain point of stretching only—*i. e.* that undue stretching is not followed by elastic recoil is well illustrated in sprained limbs, in which the tendons are not alone injured. A muscle overstretched very slowly recovers its original elasticity. But, important as is elasticity in muscle, it is more so in the tube system of the circulation. It is evident that but for this property there could be no *continuous* flow of the blood, a matter of great moment to a highly organized animal like a mammal. The immediate cause of this constant blood-flow is the elastic recoil of the arteries, which following upon distension in consequence of peripheral arteriole and capillary resistance, expends the force of the heart that had been stored up in the arterial wall during the cardiac systole.

I have been led to write briefly on this subject in consequence of having felt that there is a field in pathology and physiology in which probably most veterinary surgeons might lend assistance to clear up certain questions that arise.

One reads in the newspapers every now and then that at such and such a race-meeting a certain horse suddenly fell dead; the cause of death being stated either as heart disease or "rupture of an artery." Now it is well known that a large proportion of cases of sudden death from heart disease in the human subject, according to popular notions, is really due to other causes; while the bursting of an artery is of the very rarest occurrence, except when the coats are greatly diseased—we may say diseased in a way palpable to naked eye observation. I have not learned that the opinions expressed by the newspapers in the cases referred to among horses were based upon *post-mortem* examinations.

There are several questions to be decided in this connection. If death be due to the bursting of a blood-vessel, what sort of a vessel, vein or artery, large or small? Where is the vessel situated? In brain, lungs, or elsewhere? What the naked eye condition of the vessel concerned? Is the heart sound? Is the structure of the vessel shown on microscopic examination to have been altered? What is the age of the animal? If a vessel can be ruptured in a healthy animal simply by excessive exertion, and that vessel, by the closest examination of its structure, shows no changes the eye can appreciate, is it possible that invisible *molecular* changes of structure may be produced by the treatment of an animal as making up what is called "training," using that term in its widest sense? Can special feeding produce such changes; or if such alterations do really occur, what is it brings them about? The answers to such questions as these seem to me of great importance, scientifically and practically. But the first thing to be ascertained is the actual cause of death in these cases.

The *post-mortem* examination of every race-horse is, from many points of view, of the greatest interest, and the publication of the results in such a Journal as this might be regarded as a valuable donation ot knowledge to the entire medical profession. I shall now endeavor to indicate briefly the scientific basis on which the above questions rest—in part at least.

Some years ago a most important investigation, bearing on this subject, was carried out by Dr. Roy, then a physiologist, now professor of pathology at Cambridge, England. The principal conclusions at which he arrived in regard to the elasticity of the arterial wall may be summarized somewhat as follows:

1. The arteries in healthy animals are most distensible with internal pressures such as during life have existed in their interior. The maximum increase of capacity in the case of the aorta and larger arteries is reached with a pressure equal to that corresponding to the normal mean blood-pressure of the animal from which the specimen of arterial wall is taken for investigation.

2. The elasticity of the arteries is much more readily modified by diseases affecting the general nutrition than is generally imagined. Slow febrile processes producing marasmus may completely modify the form of the curve of elasticity. The relations of the pressures to the volumes of portions of the aorta or other large artery from human beings or the lower animals, which have suffered from wasting disease not specially affecting the arteries, is quite different from that which exists in health. When marked marasmus has existed before death the arteries are found to be relatively wider than normal, and are most distensible with internal pressures immediately above zero.

3. The effect of age on the elasticity of the arteries is very marked. Only in young children is the elasticity so perfectly adapted to the organism as in the lower animals; there is a gradual decline with the advance of years in the human subject. But in the lower animals this perfection of elasticity is found in the adult as well as in the young animal. It would appear, however, that while there is not in the circulating system of the young adult man the same capacity for ready adaptability to circumstances as in the child, still the arterial elasticity in the adult is fairly good up to 30 years of age, though not comparable to that of the lower animals.

Dr. Roy has given an account of the examination of the arteries of a dog that had been reduced to a state of extreme emaciation either from chronic illness or starvation. The examination of its vessels showed that the arterial elasticity curve was wholly different from that of healthy dogs. "The artery was more distensible the lower the intra-vascular pressure." And I would myself direct special attention to the fact that in this case *no structural change* of the walls of the vessel could be detected either by the naked eye or by careful microscopic examination of hardened specimens. Hence, the point of some of my queries, stated above, in regard to the race-horse.

When we consider that in the development of a living creature there is a constant adjustment of internal conditions to external environment one would not expect to find that in a healthy animal, rupture of a blood vessel, especially of a large vessel, and above all of an artery, should occur as any possibility of exertion—even the most severe.

It seems, however, to have been shown that the race-horse of the present day has not the stamina of his predecessor. Great speed appears to be now developed at the expense of constitutional vigor. Have the arteries become effected in this process? In the human subject it has been shown that the heart may by affected by long continued athletic strain. Is this true of the race-horse?

The secret of the most healthful and perfect life an animal can enjoy lies in the *balance of all its functions.* The more perfect our physiology, the more complete will be our insight into these relations; and to understand a disease perfectly will be to explain all disturbances of the vital equilibrium.

It would be interesting to ascertain what changes, if any, take place in the elasticity of the arteries of wild animals when kept in confinement. The friends of scientific medicine will note with pleasure that autopsies are held upon the animals dying in the Central Park Menagerie, New York.

If this brief paper serves in any way to awaken increased interest in any question of *scientific* medicine, whether that treated or others, it will have served some purpose.

